# Development of activated carbon for removal of pesticides from water: case study

**DOI:** 10.1038/s41598-022-25247-6

**Published:** 2022-12-02

**Authors:** Bartosz Zieliński, Piotr Miądlicki, Jacek Przepiórski

**Affiliations:** 1 Grand Activated Sp. z o.o., ul. Białostocka 1, 7-200 Hajnówka, Poland; 2 Engineering of Catalytic and Sorbent Materials Department, Faculty of Chemical Technology and Engineering, West Pomeranian University of Technology in Szczecin, Pułaskiego 10, 70-322 Szczecin, Poland

**Keywords:** Environmental sciences, Chemistry, Engineering, Materials science

## Abstract

The work primarily concerns development of activated carbon dedicated for adsorption of pesticides from water prior directing it to the distribution system. We provide an information on research on important practical aspects related to research carried out to develop and to manufacture activated carbons. The paper concerns preliminary works on selection raw materials, a binder used for producing granulated adsorbent, activating gases, conditions of the production process, and others. The key attention in this research was paid to its target, i.e., industrial process to produce activated carbon revealing fulfilling required properties including satisfying adsorption of selected pesticides and meeting the requirements of companies dealing with a large-scale production of drinking water. Therefore, among others, the work includes considerations concerning such aspects like pore structure and specific surface area of the activated carbon, formation of granules that are the most demanded and thus preferred in an industrial practice form of activated carbons, and other aspects important from practical point of view. Using the results of our preliminary work, a batch of granular activated carbon was produced in industrial conditions. The obtained material was tested in terms of removing several pesticides at a water treatment plant operating on an industrial scale. During tests the concentration of acetochlor ESA was decreased from ca. 0.4 µg/l in raw water to below 0.1 µg/l. During 11 months of AC use specific surface area of adsorbent lowered significantly by 164 m^2^/g, and total pore volume declined from initial 0.56 cm^3^/g to 0.455 cm^3^/g. We discuss both a performance of the obtained activated carbon in a long-term removal of acetochlor and its derivatives from water and an effect of exploitation time on the removal efficiency. The explanations for the reduction in pesticide removal efficiency are also proposed and discussed.

## Introduction

Freshwater reserves are the basic resource for all life on our planet. Climate change and irresponsible hydrological management have led to a significant reduction in the amount of easily available water^1^. This serious problem concerns many regions including Europe. Poland is one of the countries of Europe with the smallest water resources per capita and is endangered with a shortage of utility and drinking water. Long-lasting and frequent droughts reduce the flows and water levels in streams and rivers, which considerably increases the concentration of polluting agents in both surface and underground waters^2^. Moreover, industrial effluents and intensive agriculture are other factors providing pollution and thus causing poor condition of available water resources. In effect, natural water resources, especially surface waters, are contaminated with various chemicals, metals, suspended matter, nutrients (nitrogen and phosphorus), pathogens, pesticides and herbicides^3^. Water pollution caused by pesticides and herbicides is a well-known serious environmental problem with undesirable effects on human^4^ and animal^5^ health.

### Pesticides and problems related to their use

**Figure 1:**
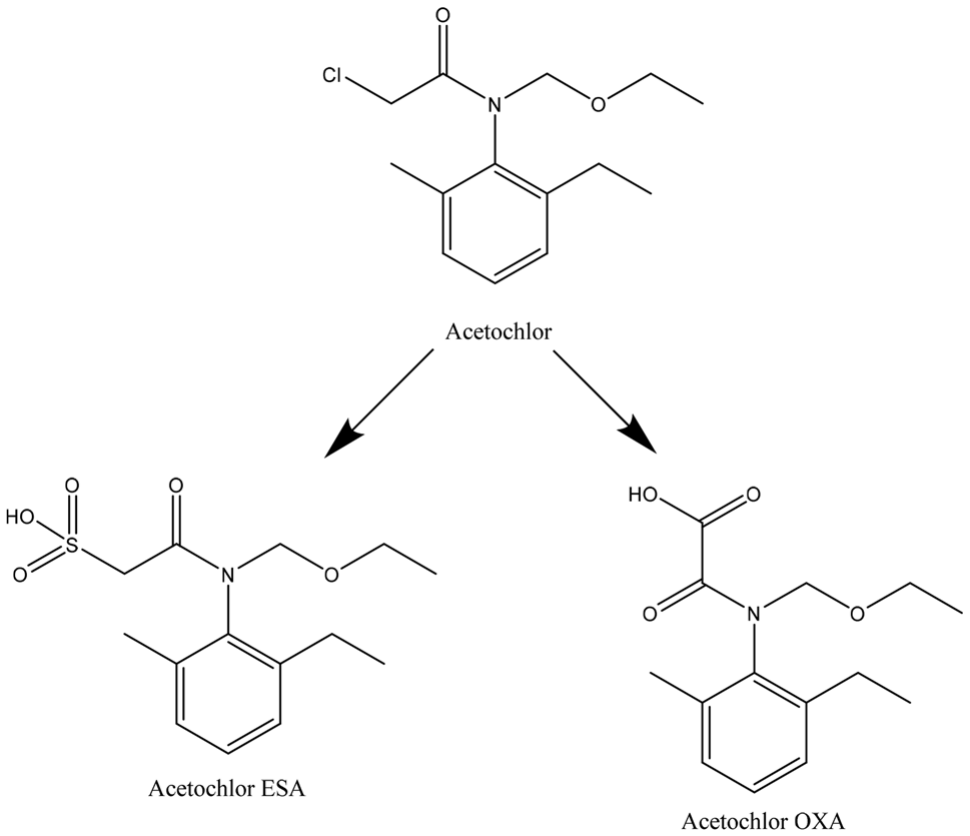
Chemical structure of acetochlor and their degradation products: ethane sulfonic acid (ESA) and oxanilic acid (OXA).

The most frequently detected pesticides in waters are those that have been used extensively and typical example acetochlor (2-Chloro-*N*-(ethoxymethyl)-*N*-(2-ethyl-6-methylphenyl) acetamide)^6–8^ and its metabolites^9^. This popular herbicide is used for the control of most annual grassy weeds and some annual broadleaf weeds. Crops treated with acetochlor include cabbage, corn, potatoes, strawberries, cotton, green peas, onion, soybeans, sugar beets, and vineyards^3^. In Europe, the herbicide is used mainly for corn treatment. A half-life of acetochlor is 5–25 days^10^. During this period, it undergoes degradation in soil and water into metabolites—oxanilic (OXA) and sulfonic (ESA) acids (Fig. 1)^11^.

Acetochlor has been classified as a probable human carcinogen^12,13^. Moreover, the USEPA and European Environmental Agency recognized this chemical as an endocrine disrupting agent. Because of important problems related to exploitation of pesticides in general, many efforts are made to remove these harmful compounds during water treatment.

### Methods used for removal of pesticides from water

Several approaches reveal a potential to remove pesticides from water. These include advanced oxidation processes like Fenton’s reaction, photochemical degradation, chlorination, biological treatment and membrane filtration^14^. The advantages and disadvantages of each of the different techniques are presented in Table 1^15–19^. However, the most efficient and the most widely used way to arrest pesticides contained in water streams is adsorption on activated carbons^20^. This is due to their large surface area, high adsorption capacity and some content of surface functional groups—often giving to the carbon surface properties enhancing adsorption. For these reasons, efforts are being made to tailor both pore structure of activated carbon and surface chemistry of the material. This can be achieved either by carrying out production process under purposefully selected conditions or by applying various modifications of the product. Regardless of the manner used, the target is obtaining adsorbent being highly effective in removal from water various chemicals including pesticides^21^.

**Table 1:** The advantages and disadvantages of technologies in the removal of pesticides from water.

Method	Advantages	Disadvantages
Fenton oxidation	Effective in treating pesticides contaminated wastewater on the industrial scale. Degradation and mineralization of pesticides. Established technology	Sludge formation. Operation in acidic conditions (pH 3) and requires neutralization of pH
Chlorination	Relatively simple maintenance and operation. Inexpensive. Easily available	Use of highly corrosive agents. Has odour and taste concerns. Formation of by-products
Photochemical degradation	No sludge production. Fast treatment. Solar energy can be used. Established technology	Formation of by-products. Energy and cost intensive process. UV penetration problems
Biological treatment	Economical, high efficiency, environmentally friendly, regeneration not required. Complete mineralization of contaminants to CO_2_ and H_2_O without build-up intermediate	Decomposition and degradation of biosorbents. It is not effective when the growth of microbes is not supported by environmental conditions
Membrane filtration	Small space requirement. Very high efficiency	High investment and operational cost. Membrane fouling problems
Adsorption: use of adsorbents	Easy and cheap to implement. Cost effective and high efficiency. Wide availability of various sorbents	Pesticides are not being destroyed but are being transferred from one medium to another. Regeneration difficulties

### Activated carbons: considerations on use and production

#### Effect of pore structure on the adsorption by activated carbons

The pore size distribution (PSD) of activated carbons is one of the most important feature affecting the adsorption process. The PSD determines the fraction of the total pore volume that can be accessed by an adsorbate of a given size^22^. During purification of freshwater synthetic organic chemicals (SOCs) and dissolved natural organic matter (NOM) compete in adsorption in the pores of activated carbon. NOM consists of small molecules with low molecular weight, such as carboxylic and amino acids and proteins. Due to relatively high concentrations in waters, the NOM competes directly with SOCs (pesticides, algal toxins, taste and odour compounds) for adsorption sites^23^ especially in micropores. On the other hand larger molecules of humic and fulvic acids—were confirmed to be adsorbed in mesopores and macropores^24^, thus blocking access to micropores for other adsorbates. The size of the acetochlor ESA and OXA molecules at its widest point is about 10.5A, so adsorption in micropores close to 2 nm is possible.

The microporous nature of activated carbons promotes the adsorption of adsorbates of low molecular weights; however, adsorption of large molecules needs an adsorbent containing larger pores including mesopores and sometimes macropores. Moreover, given the size of the pesticide molecules, adsorption energies are greater in micropores. In addition, due the greater number of contact points between the molecule and the adsorbent, preferential adsorption is attributed to the molecules similar in size to pores. The overlapping potential forces created when opposing pore walls are separated by little more than the diameter of an adsorbed molecule is responsible for the increased adsorption forces in micropores^22,25^. It should also be taken into account that the presence of only micropores can seriously affect them performance in the respective processes due to the extreme slow mass transfer through micropores. In order to improve the transport properties of microporous materials, the conditions of the preparation process are selected in such a way as to include the mesopores in the micropore space as additional, low-resistance transport routes^26^.

The iodine number is often used as a simplified method to estimate the volume of micropores in activated carbons being produced^27^. The correlation between the amount of iodine adsorbed by an adsorbent and the volume of micropores determined by using more advanced methods, has been confirmed as satisfactory and sufficient for use in a common industrial practice.

#### Effect of surface chemistry od activated carbon on adsorption pesticides

Application of activated carbons is highly dependent on the surface chemistry. The use of activated carbons with oxygen surface groups in the case of adsorption from water is ineffective. It is related to the absorption of water by hydrogen bonds to the surface oxygen groups, which causes clogging of the pores and limits the adsorption of organic compounds^28^. In the pesticide adsorption on carbon materials the main force is expected to be the dispersion force between the π electrons in the pesticide structure and the π electrons on the surface of carbon material^29^. Electron-rich Lewis base sites are formed when oxygen-containing functionalities are removed from the surface of the activated carbon. Removal of oxygen not only makes the surface of the activated carbon more basic, but also less polar. These properties are desirable in organic adsorption from aqueous solutions^30,31^. In order to obtain activated carbon without surface oxygen functional groups, activated carbon must be modified by hydrogen treatment^28^ or thermal treatment in an inert gas atmosphere^30^. Under industrial conditions, modification or controlling of the surface chemistry of activated carbon by mean of content of oxygen—containing functional groups would be difficult and costly, especially at large scale production. For these reasons, conventional industrial production of activated carbons does not involve treatments aimed on controlling content of surface oxygen functionalities in a product.

#### Production of activated carbon

Key factors influencing on the structure and properties of the produced activated carbon and the most important are a kind of raw material, composition of activating agent, durations and temperatures of carbonization and activation processes, or in the case of chemical activation process—ratio of activating agent to carbon material^32,33^. The most popular raw materials for activated carbons production are coals, wood, coconut shells, and sometimes peat^34^. Due to beneficial combination of availability and balance between manufacturing costs and performance, coal—made adsorbents are the most commonly used in water treatment, chiefly for removal of organic contaminants including wide spectrum of pesticides. Besides the conventional raw materials, alternate ones have been proposed to produce activated carbons. Here, the most frequently reported precursors for activated carbons are certain polymers^35–39^ and pitches^40–45^, or a wide range of other organic materials^46–53^. Numerous reports concern use of these unconventional adsorbents for adsorption of pesticides from water. However, in spite of quite intensive research, these materials have not found wider interest in industrial reality.

The adsorption of pesticides from water is always timely topic. Vukcevic et al. obtained activated carbon from waste hemp and used it for adsorption of acetamiprid, dimethoate, nicosulfurone, carbofuran, and atrazine. The preparation of activated carbon was carried out in two separate stages. As first the raw material was subjected to carbonization at 700 or 1000 °C, and then activated with KOH at 900 °C under nitrogen flow. The obtained activated carbons revealed dominantly microporous structure and high specific surface area (up to 2192 m^2^/g). The authors found the highest efficiency in pesticides removal as dependent on amount of KOH used for the activation process. Moreover, it was found that the amount of KOH is a factor influencing the amount and type of oxygen functionalities on the surface of activated carbon^52^. Microporous activated carbon with specific surface area of 981 m^2^/g obtained from waste tires was reported as suitable for removal methoxychlor, methyl parathion and atrazine from water. The average pore diameter calculated for the adsorbent was 3.12 nm. And in the fixed bed adsorption 70–90% removal of the pesticides could be achieved^44^. The adsorbent was obtained from ground tire granulate in three-step process consisting of carbonization conducted at 500 °C for 5 h, treating with hydrogen peroxide to oxidize organic impurities^45^, and activation using KOH at the temperature of 900 °C for 2 h. Hydroxyl and carbonyl groups formed during the activation process on the carbon surface were confirmed as favouring adsorption of the pesticides. Effective and rapid removal of pesticides from water was reported also for mesopore—rich activated carbon, prepared from starch through chemical activation process using Na_2_CO_3_ and KOH. The pesticide adsorption was confirmed as strongly dependent on the electron donating abilities of the oxygen-containing functional groups and adsorption increased with amount of oxygen functional groups on the surface^53^. It should be noted that in spite of quite intensive research carried out at numerous laboratories a role of the unconventional raw materials in large scale production of activated carbon is minor. In industrial practice, economical aspects are of the top importance. Therefore, selection of raw material for production of activated carbon is a key decision to be made by a producer. In production of most of the commercially available activated carbons various coals (anthracite, bituminous, sub-bituminous and lignite), wood and coconut shells, serve as raw materials. A small share in the production of specialized activated carbons is taken by peat, waste cellulose materials, olive stones, petroleum coke, pitches, or synthetic polymers and Ref.^54^. The properties of ACs such as: specific surface area, pore volume, pore size distribution, surface chemistry, mechanical resistance, and particle size of GAC can be customized according to the final use of the ACs by changing the producing process parameters. In industrial reality, most of activated carbon is produced using so-called physical activation process. Conventionally a gas composed of steam and carbon dioxide is used as an activating agent. Such a manner is preferred due to attractive economical effect and favourable technological features including scalability and lack of difficult to manage wastes^55^. There are several factors influencing on properties of the manufactured activated carbon. Besides the type of raw material used, these include composition and flow rate of the activating gas, temperature, and duration of the process.

Increasing the flow rate causes acceleration of gasification rate and discharging the volatile matter from the surface of carbon precursor subjected to the activation process. Besides that, at high flow rates temperature of the reaction zone and contact time between the activation gas and the activated material is normally reduced. The use of sole CO_2_ as an activating gas, favours the formation of a larger, compared to the process carried out with use of steam only, volume of micropores. Reactions of both CO_2_ and steam, with the carbonaceous material are endothermic and therefore the activation temperature substantially affects predominantly yield and also porosity (pore volume and specific surface area of the obtained activated carbon. For that reason, a manufacturer of a material must decide, taking into account properties of a raw material used, which temperature is optimal to run production process economically.

For coal-originating activated carbon precursors, the temperature typically used for the activation process ranges from ca. 700 to ca. 950 °C^56–58^. Below the lower limit the process does not proceed efficiently due to low rates of activation reactions. On the other hand, the process carried out at temperatures exceeding the upper limit is accompanied by a rapid and therefore hard to control gasification of the carbon material by the activating gases. Another potential effect accompanying activation at the high temperature regions is undesirable destruction of the interpore walls and thus conversion of micropores to larger ones and by overall lower yield of the process. Analogous effects are attributed to the duration of the activation process. It is well known that excessive exposure of a carbon material to the activation agents results in both obtaining activated carbon revealing relatively low content of micropores and in high burn-offs^57^ affecting economics of the production.

#### *Effect of granulationon the adsorption process*

In the water treatment process, activated carbon can be used in two forms, i.e. as powdered activated carbon (PAC) and granular activated carbon (GAC). For most commercially available PACs, the particle size does not exceed 44 µm. This form of activated carbon generally performs very efficiently and is mostly used for accidental pollution removal lasting up to several days or for random contamination of the water, primarily with organic substances. In most cases PAC used in such a manner cannot be economically recovered from the treated water stream, so it is routinely disposed of with process solids^59–61^.

In water treatment, GAC is most often used kind of activated carbon. It is usually manufactured either in the form of crushed activated carbon grains, or as granules. Granules or other shapes like small cylinders are normally obtained through either pressing or extrusion, from a dense paste consisting of a raw material and an organic binder. Binders such as gas tar, beet molasses, tall oil, humic acids, or adhesive cellulose and sometimes clays are used to obtain the paste. Due to the variety of physical and chemical properties of the potential binders, features of produced GACs are not constant. It is demanded the binder is not removed from the material being treated and during the production process it undergoes conversion to carbonaceous material and finally becomes an activated carbon. Additionally, the binder is expected to provide to the GAC suitable mechanical properties and to be chemically resistant^62,63^. Utilization of activated carbons is strongly affected by adsorbent’s grains size and in the case of granular activated carbons, the grain-size distribution affects adsorption kinetics^64^ substantially affecting adsorber’s performance. The GAC grain size is determined at the early stages of the production process, i.e., during extruding the mixture of raw material with the binder or by crushing the raw material. In general, small GAC grains reveal superior adsorption kinetics and capacity, however, they may cause substantial pressure drops in the filtration beds during operation. On the other hand, larger GAC grains usually exhibit a lower adsorption capacity per weight. Such effect is due to the relatively small pore volume of such material, resulting from less efficient activation of the cores of large grains. Hence, selection of activated carbon strongly depends on the needs and technical requirements, including scale, target adsorbates, and yield^59^.

Typical size of GAC grains supplied by industry ranges from 0.2 to 5 mm^65^. This commonly exploited adsorbent is normally used in packed bed filters, principally for the removal of organic compounds from water streams being treated^25,65^. Using activated carbon (GAC or PAC) together with a coagulant result in lowered adsorption efficiency of the adsorbent. Such effect is due to binding of activated carbon to the flocs formed during the process that deteriorates the diffusion of an adsorbate to the adsorbent’s pore system. Therefore, in the drinking water production, activated carbons should be used to treat water after coagulation and filtration.

An important practical advantage of GAC is the possibility of its regeneration, normally carried out to restore the adsorption capacity of the material after use. Several regeneration methods are known. These employ chemical reactions^66^, electrochemical processes^67,68^, and oxidative^69^ and thermal^70^ treatment. Other, less popular methods used for regeneration of activated carbon employ microwave^71^, plasma^72^, or biological^73^ treatments. Not all of them have found an interest in industrial practice. Nevertheless, regeneration in the industrial scale must improve an overall economic effect of the application.

### Conclusions and practical recommendations for manufacturing activated carbon for adsorption of pesticides from water

From practical point of view, there are several important aspects to be taken into account during designing and choosing activated carbon for specific use. This concerns also targeted application like removal of pesticides from water streams, which process is usually carried out in a continuous mode and with used of filtration beds. The latter determines the recommended and preferred form of an adsorbent—GAC. Nevertheless, even GAC is chosen for this application, the end used should consider and examine which granule sizes give an expected performance of the filtration system. Hence, while fine granulations may cause an undesired and often considerable pressure drops during water filtration, an access of the adsorbate to the pore system offered by a coarsely granulated adsorbent is limited. Another important parameter is hardness. This mechanical feature is directly connected with a possibility of loss—free regeneration when the spent activated carbon is being thermally treated to desorb primarily organics from GAC and thus to recover a pore system for another use. In the process, hardness of the AC is essential for limiting losses of the carbon during reactivation. In industrial practice generally accepted optimal granulation of activated carbon is from 0.5 to 2.5 mm. The range includes granulation from 1 to 1.5 mm, that has been found by the Grand Activated as fulfilling technological expectations.

Another important aspect is content of elements other than carbon in the AC. Here, a high level of ash in activated carbon is not desirable in terms of the use value of the product. However, the presence of certain compounds in the raw material may reveal a noticeable effect on the production process and characteristics of the products. For example, presence of potassium in carbon structure results widen space between graphite layers and is leading to creation of larger size of pores volume^74^. Nonetheless, washing out of non-carbon components from an activated carbon with water or acids is a normal practice used if necessary.

Based on the above, we can propose a reasonable materials and process to produce activated carbon for removal of pesticides from water streams. Because of an attractive price and low level of impurities, it seems reasonable to use a quality hard coal as a raw material to manufacture activated carbon of expected properties. Because of readily availableness and beet sugar molasses may be a good option as a binder to form shaped grains for further treatment. Because a chemical activation process is accompanied by generation of chemically polluted streams that requires an extra treatment, a conventionally used physical activation using steam and carbon dioxide seem to be the optimal option for treating raw carbon precursor at elevated temperature to carry our both carbonization and subsequent activation. Moreover, it can be expected that GAC obtained with use of the gases will show a high content of micropores with the presence of mesopores—and the latter is a typical effect of certain content of inorganics in the carbon precursor subjected to the carbonization and activation processes. In addition, proposed raw materials are known to give a mechanically resistant activated carbon, with a real potential for long-term exploitation and treatments such as washing, transportation and regeneration. Because textural properties of activated carbon are normally affected by the parameters of the process it is necessary to carry out research on the effect of activation temperature, composition of the activating gas, and duration of the process on the product of interest—activated carbon. This work is intended to lay the foundations for the implementation of a new type of activated carbon in a company product range, for the removal of pesticides from drinking water. The aim of developing such a product is to meet rapidly growing market demand. We plan to use sustainable raw material (molasses) in the production and try to limit generation of waste.

## Experimental

The development of a new material requires preliminary research from the selection of raw materials through to production trials and testing.

### Selection of raw materials

The carbon precursor of our choice was bituminous coal supplied by a Polish mine. This coal reveals low content of ash (Tables 2, 3). Despite the composition of ash (content of metals such as K, Na, Ca) influences the formation of pore structure during activation^75^. Excess of this component increases rate of the activation reactions and thus makes controlling of the production process very problematic due to real risk of obtaining not satisfactory quality and low yield. Therefore, this carbon precursor was considered as suitable candidate material for manufacturing of highly microporous, and thus appropriate for adsorption of pesticides, material with rather small content of both mesopores and macropores.

**Table 2:** A proximate composition of bituminous coal used for preparation of activated carbon.

Volatiles content (wt% of dry mass)	Moisture content (wt%)	Ash content^a^ (wt%)	Sulphur content^b^ (wt%)	Phosphorus content (wt%)	Cfix^b^ (wt%)
35.00	5.12	5.55	0.5	0.04	53,79

^a^Ash content of the coal considered as the candidate material for manufacturing of AC is shown in the Table 2.

^b^Fixed carbon content.

**Table 3:** Average contents of main ash components in coal selected for preparation of activated carbon.

Content (wt%)								
SiO_2_	Al_2_O_3_	Fe_2_O_3_	CaO	MgO	Na_2_O	K_2_O	TiO_2_	P_2_O_5_
32.86	26.7	13.4	7.95	4.41	2.28	1.56	0.8	1.26

The binder material chosen was the sugar beet molasses obtained from a Polish sugar factory. Properties of the molasses are listed in Table 4.

**Table 4:** Properties of the sugar beet molasses used for preparation of activated carbon.

pH	Moisture content (wt%)	Sugar content, (wt%)	Cont. of others (amino acids, inorganic other), (wt%)	°Bx	Viscosity, cP					Content of main components in ash					
					20 °C	30 °C	40 °C	50 °C	55 °C	Mg (mg/g)	K (mg/g)	Na (mg/g)	Ca (mg/g)	Fe (mg/g)	Carbonates, (wt%)
8.77	22.1	44.8	33.0	79.3	6791	2511	996	453	317	0.1	37	11	0.46	0.039	10

°Bx (Degrees Brix)—a measure of the amount of dissolved sugar in water. 1°Bx means 1 g of sucrose in 100 g of aqueous solution.

The molasses is commonly used as a binding material to produce granular activated carbon (GAC)—a regular type activated carbon. It should be noted that the molasses normally contains a small but noticeable amount of potassium. Because compounds of this element are known chemically activating agents^74,76,77^, action of potassium compounds on the material being activated may be expected next to the predictable processes involving CO_2_ and steam. Normally, to produce a shaped raw material for manufacturing a coal-based GAC, 5–35 wt% content of molasses is used in molasses/coal mixes^62,78,79^. In practice, the content of molasses in the mixes is decided after taking into account desired textural properties and mechanical strength of the GAC. Typically, excessive levels of a binder do not favour obtaining highly porous activated carbon. However, high contents of a binder in raw material promote obtaining GAC revealing relatively high mechanical strength^80^. Considering above we have decided to use molasses/bituminous coal paste composed of 18% of the first and 72% of the latter.

### Step by step preparation process

**Figure 2:**
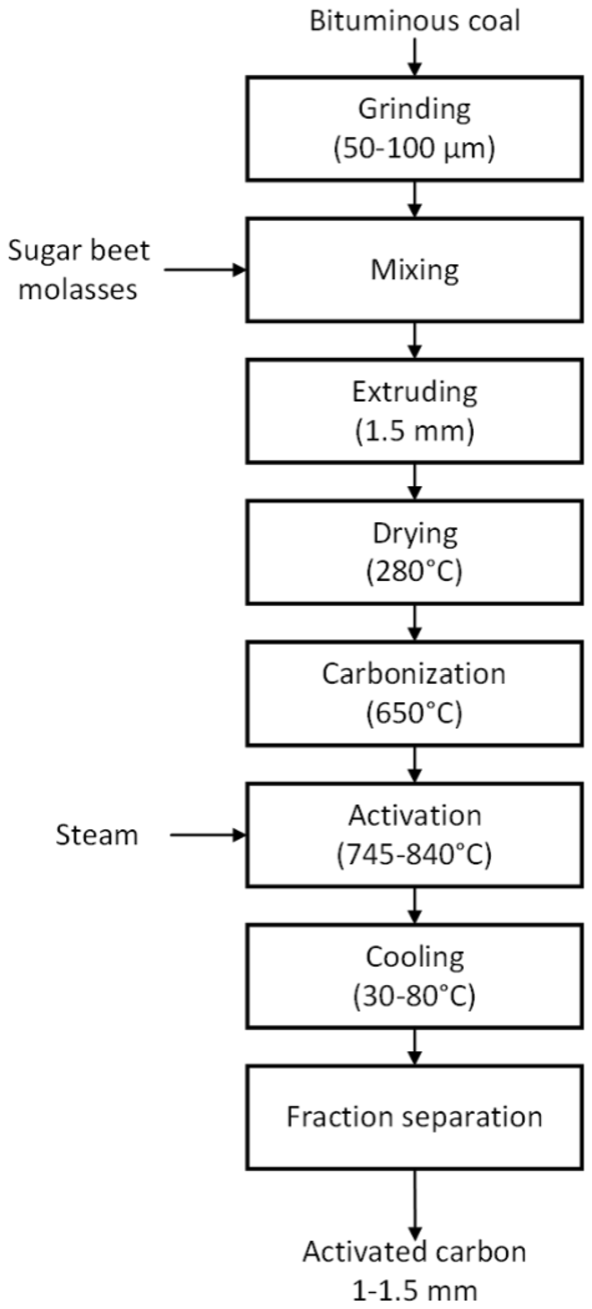
A scheme of preparation route used for obtaining activated carbon.

The activated carbon developed in this work was preparation process of obtained according to the procedure schematically shown in Fig. 2.

As first, bituminous coal is ground in a ball mill to obtain grains sized from ca. 50 to ca. 100 µm. Subsequently, sugar beet molasses in an amount of 18 wt% is added to thus prepared powdered coal followed by thorough mixing to obtain homogeneous dense mass. Then, the obtained mass is directed to an extruder to prepare cylindrical pellets of 1.5 mm in diameter 5 mm in length. In normal conditions of the production hall ca. 0.5 Mg/h of the coal are processed. The obtained shaped grains are then placed in a rotary kiln at 280 °C and dried with the air to a moisture content below 1 wt%. As next, the dried material is subjected to lasting 2 h carbonization process carried out in a hermetic rotary furnace under slightly reduced pressure (− 0.10 kPa) at 650 °C. The flue gases that are released during the process are consumed in the plant's energy management, including for the operation of the process, and are burnt in order to maintain necessary temperature inside the furnace. The char obtained during carbonization contains up to 3% of volatile matter and the mechanical strength of the carbonization product is at least 95%. The activation process is carried out in a rotary furnace in the atmosphere of steam under a slightly lowered pressure, forced by a fan installed at the outlet of the kiln. Steam is an efficient and relatively cheap activating agent and therefore is commonly used by activated carbon manufacturers. Steam (ca. 0.5 Mg/h) is supplied to the furnace through a nozzle. The temperature and duration of the activation is 785–840 °C and 3 h, respectively. At the outlet of the rotary furnace the hot activated carbon is cooled (through discharging it to the excess to demineralized water), filtrated and finally dried in an oven (30–80 °C) to the moisture content of ca. 10%. In order to obtain the assumed granulation, the dried activated carbon is sieved with a mesh to separate grains of 1–1.5 mm in size.

### Methods

#### Characteristics of the activated carbons

Nitrogen adsorption/desorption at 77 K isotherms were measured to determine textural properties. QUADRASORB evo Gas Sorption Surface Area and Pore Size Analyzer was used for that purpose. The Brunauer–Emmett–Teller (BET) model^81^. was employed to calculate the specific surface area of the activated carbon being developed. The total pore volume (TPV) was estimated from the volume of N_2_ adsorbed at p/p_0_ ≈ 1. The density functional theory (DFT)^82^ method was used to calculate of the micropore volume (MPV) and pore size distribution (PSD). Before N_2_ adsorption/desorption isotherms were measured, the materials were degassed at 200 °C under deep vacuum. Other parameters of the studied materials were determined in accordance with the standards as listed in the Table 5.

**Table 5:** Standards used for measuring parameters of the developed activated carbons.

Parameter	Standard
Iodine number	PN-83 C-97555/04
Mechanical strength	PN-90 C-97554
Bulk density	PN-90 C-97554
Ash content	PN-84 C-97555/08
Moisture content	PN-84 C-97555/09
Volatile parts	PN-G-04516

#### Method used for determination of the pesticides’ concentrations in water

Activated carbon chosen for pesticide adsorption was tested in real conditions on a drinking water production plant. Concentration of a pesticide in water was measured using liquid chromatograph coupled with mass spectrometer equipped with a triple quadrupole (Agilent 1200 system ZORBAX Extend-C18 column mounted, with MS detector 6120 SQ). The site’s ambient temperature conditions were stable for optimum performance. Total samples of 100 µl were used and the LC/MS apparatus was calibrated using reference sample concentration of 0.5 µg/l of determined compounds.

## Results and discussion

Properties, those required by end users of the product, of the activated carbon produced according to the procedure described in “Methods”, in various production parameters, were examined. The most promising AC was chosen and subjected to testing on existing technical equipment.

### Effect of preparation conditions of characteristics of activated carbons obtained

By tracing the graph of the dependence of the amount of material obtained on the iodine number obtained, the yield decreases as the activation level increases. This is due to the decrease in the density of the carbon skeleton through the expansion of the pores. The decomposition of coal structure is confirmed by the plot of bulk density vs. iodine number. The higher the activity level (iodine number) the lower the bulk density (see Fig. 4).

**Figure 3:**
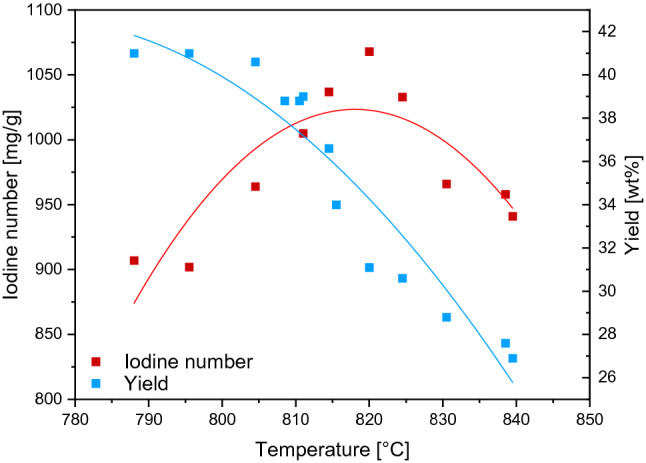
Relation of activation temperature and the yield of activated carbon, and iodine number.

Figure 3 shows the relationship between the temperature of the activation process, recorded with use of a thermocouple placed in the oven shell at the midpoint, and the yield and the iodine number of the product. From the graph, it can be concluded that setting the temperature in the range of 805–830 °C one is obtaining the maximum Iodine Number level in the given production conditions. When temperature exceeds 810 °C yield drops sharply. To carry production in temperature higher than 810 °C will cause economic loss. The material becomes increasingly porous and the proportion of weight to volume decreases^25^. The loss of mass is an economic loss because the marginal increase in activity is less than the marginal decrease in mass and customers are not willing to cover the price. Temperature ca. 800 °C seems to be optimal for production of acceptable yield and good adsorption properties (Iodine number 1000-1050).

**Figure 4:**
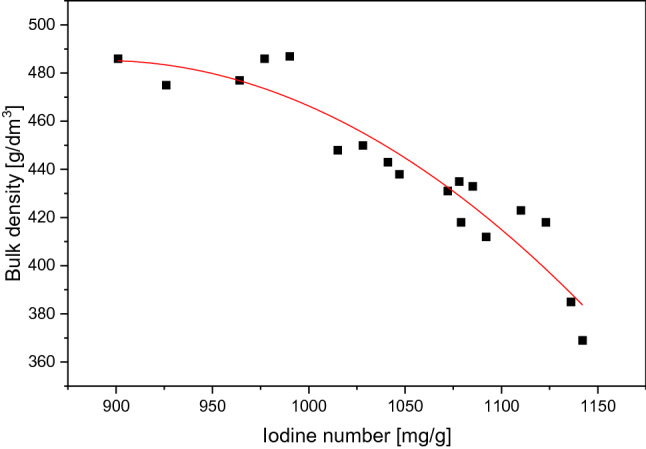
Properties of the AC prepared at various conditions-relation between the iodine number and bulk density.

A clear correlation between iodine number and bulk weight can be read from Fig. 4. It is related to pore expansion and decomposition of the carbon skeleton^75^. A decrease in bulk density—one can assume—will be associated with a mechanical weakening of the activated carbon granules and will affect the service value of the adsorbent. Conducting the process at a temperature of approx. 800 °C is expected to be proper to obtain a material with a bulk density from the range (from ca. 420 to ca. 500 kg/m^3^) accepted by the industry.

### Preparation of activated carbon dedicated for pesticide removal from water-recommendations on the basis of gained results

Taking into account both literature reports and results of research done by us, precising recommendations for activated carbon preparation can be done. Hard coal is a good candidate to be used as a precursor for activated carbon due to the fact that its activation gives good results, is a raw material (compared to others) available and cheap. We propose molasses as a binder because it allows for the agglomeration of hard coal. The high mechanical strength obtained thanks to molasses enables long-term operation of the bed (low losses due to crushing and abrasion of AC grains) and multiple regeneration. Moreover, because coal and molasses contain some impurities acting as activation catalysts^75^. It is not insignificant that molasses is a by-product of agricultural production, so it is a renewable resource and therefore is sustainable. According to our experience grain size in range 0.5–2 mm and form of regular granules ensure water flow without excessive pressure losses and even work of the whole bed volume. It is commonly known that powdered activated carbons (PAC) reveal superior adsorption parameters compared to granulated activated carbons (GAC). However due to unacceptable pressure drops former ones do not find wider application in an industrial practice as fixed filtration bed^65^. For that reason, we took decision to prepare AC in such form promising good filter bed performance and user satisfaction.

Referring to the previously presented research results, we suggest conducting the process in temp. 800 °C ± 10 under steam flow. We anticipate that the combination of the above-mentioned parameters will allow us to obtain a product with attractive parameters and will permanently enter the company's assortment.

### Pesticide removal tests with use of activated carbon developed

**Figure 5:**
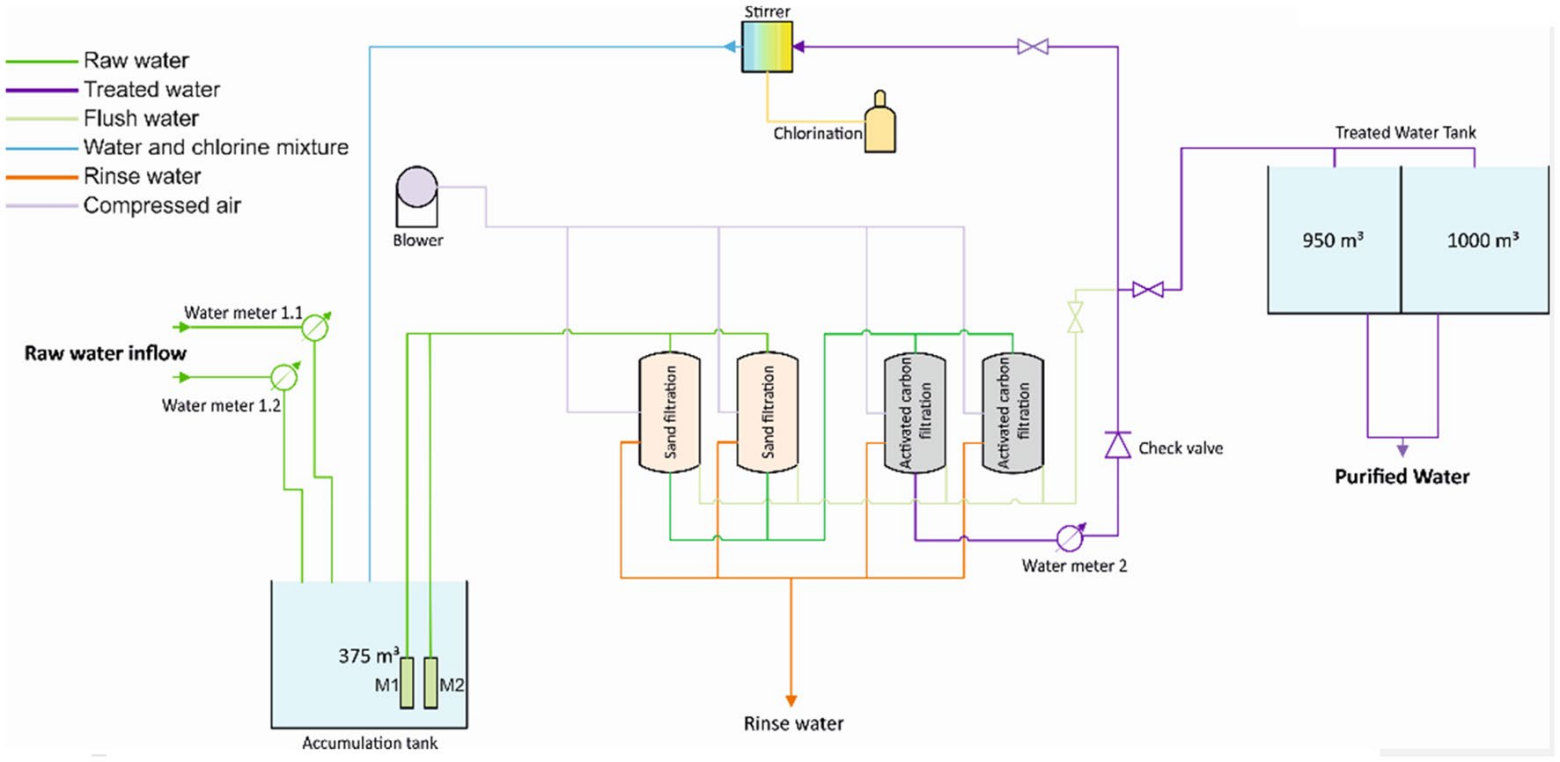
Scheme of the water filtration system.

A batch of material produced taking into account the above-mentioned recommendation (“Preparation of activated carbon dedicated for pesticide removal from water-recommendations on the basis of gained results”) was applied in the water purification process. The installation diagram is presented in Fig. 5.

Raw water is sourced from 3 different wells. Water is mixed in purpose to obtain stable water quality in the process. Flow velocity of the water was 14 l/s. In raw water 14 different pesticides/metabolites (Table 6) were detected, 4 of them were relevant, 2 of them were critical, above hygienic limit:

**Table 6:** Pesticide compounds detected in the treated waters.

Compound	Concentration
Acetochlor (ESA, OXA)	Above legal limit
Propachlor (ESA, OXA)	Close to legal limit
Alachlor (ESA, OXA)	Not relevant
Metolachlor (ESA, OXA)	Not relevant
Metazachlor (ESA, OXA), dimetachlor ESA	Not relevant
Chloridazon-desphenyl, chloridazon-methyldesphenyl, chloridazon	Not relevant

*ESA* ethanasulfonic acid metabolites, *OXA* oxanilic acid metabolites.

To remove pesticides from the water one of two sand filters body was fulfilled with 12 m^3^ of activated carbon. Changes in performance of activated carbon bed during 11 months of exploitation are described below.

### Long-term pesticide removal in an industrial scale

**Figure 6:**
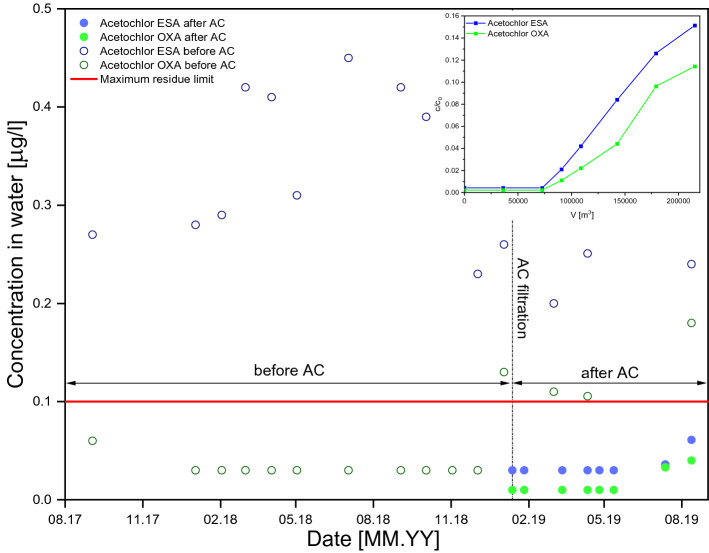
Pesticide concentration before and after the application of AC activated carbon.

Concentration of Acetochlor ESA and Acetochlor OA in raw water was detected, concentrations of the first compound were recorded above the allowable limit, concentrations of the second compound were below the limit, but trended upward and concentrations exceeded the limit in early 2019. The application of activated carbon reduced the concentrations of both compounds below the allowable limit. Despite that after approximately 6 months, the concentrations of Acetochlor ESA and Acetochlor OA in water filtered on activated carbon began to increase as can be seen in the breakthrough curve (see Fig. 6), activated carbon bed removed the acetochlor compounds satisfactorily, the concentrations after filtration were below the limit.

In order to clarify the reasons for this, the material—reworked activated carbon—after 11 months of operation, was analysed to identify changes in the parameters of the carbon (“Pesticide removal tests with use of activated carbon developed”).

### Changes in characteristics of activated carbon after 11 months exploitation

The low-temperature nitrogen adsorption isotherms for the virgin and used activated carbon are compared in Fig. 8. The isotherms appear to become a combination of types I and IV according to IUPAC classifications with type H4 hysteresis loop^83^. Table 7 summarizes textural parameters for the activated carbons. After 11 months of continuous operation, a slight decrease in the specific surface area and pore volume can be noticed.

**Table 7:** Textural parameters for the AC activated carbons before and after 11 months exploitation.

	Iodine number (mg/g)	BET (m^2^/g)	V_por_ (cm^3^/g)	V_micro_ (cm^3^/g)	Volatile parts (%)
AC	1059	976	0,560	0,301	5,49
AC after 11 months of filtration	882	812	0,455	0,275	10,38

**Figure 7:**
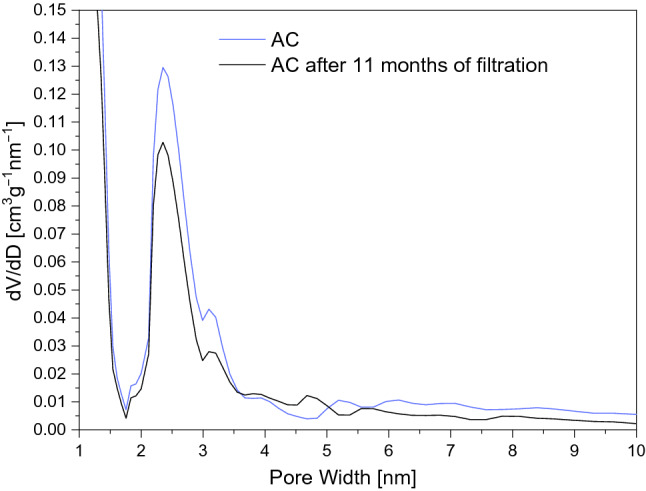
Pore-size distributions calculated for activated carbon before and after 11 months exploitation.

Figure 7 shows the pore distribution of pristine and used activated carbon. A significant reduction (about 30%) in the number of mesopores can be noticed. This is due to well known the adsorption of larger molecules (humic and fluvic acids and other compounds) during water treatment. This significantly limits access to micropores and reduces the pesticide removal rate from the water.

**Figure 8:**
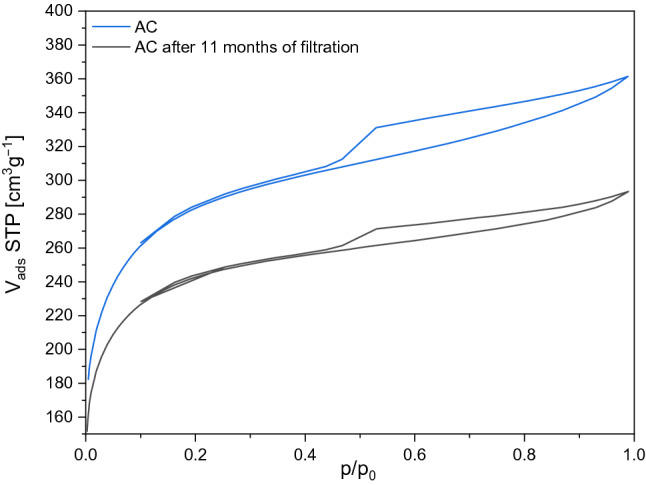
N_2_ adsorption–desorption isotherms of AC activated carbon before and after 11 months exploitation.

On the Fig. 8 It can be observed that the pore volume of the fresh coal was larger than that of the coal after filtration. In the range between 2 and 3 nm, the decrease in pore volume was the greatest.

**Figure 9:**
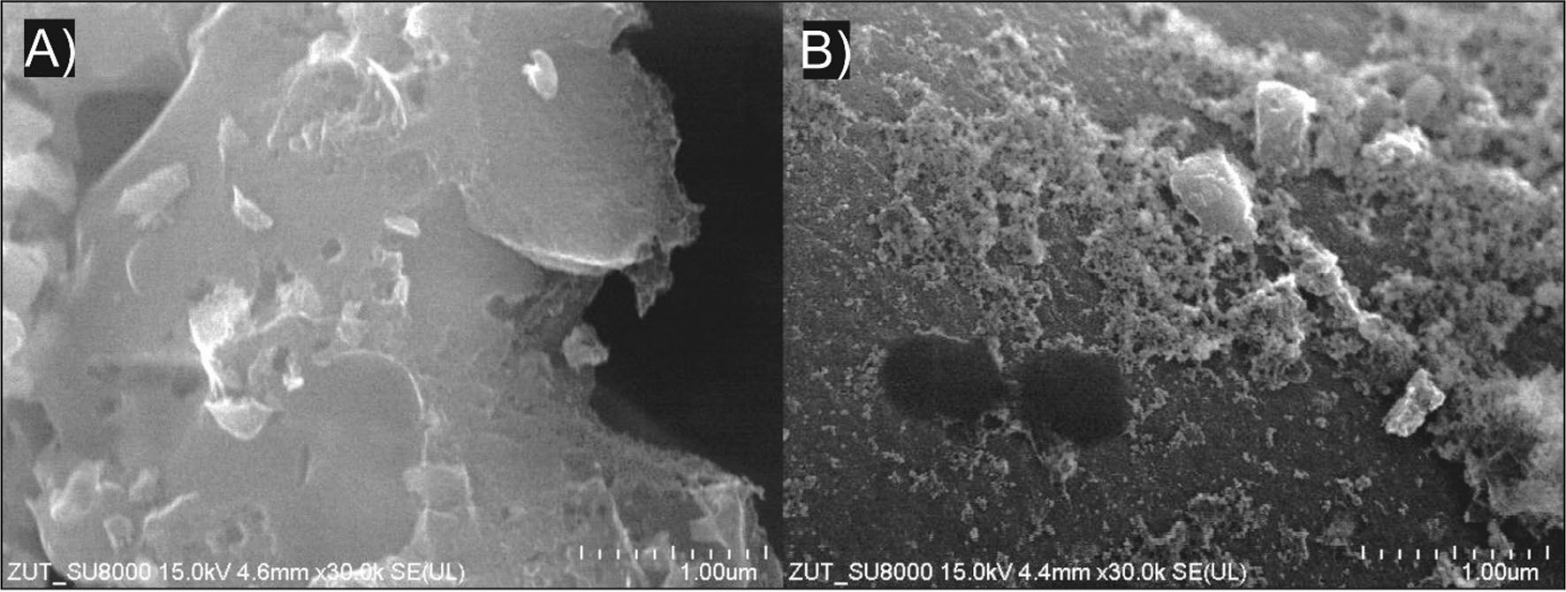
SEM images of the activated carbons before (**A**) and after (**B**) 11 months exploitation.

Figure 9 shows SEM pictures of activated carbon before and after 11 months of exploitation. A typical structure for this type of material can be seen^84^. In addition, sediment can be seen on the AC surface after use as filter bed. The resulting sediment may result from the absorption of humic acids and other larger particles and the formation of a biofilm.

## Conclusion

In this work, we described how to develop activated carbon for a specific application, i.e. to remove particular pesticides from water. In the process of development, both literature reports and many years of own industrial experience gained were taken into account. In effect we were able to decide how to step by step proceed with the selected precursor to obtain a product with parameters expected by the market and dedicated to a specific application. Our first trials resulted in obtaining a lot of an adsorbent with satisfactory commercial properties and able to remove contaminants (Acetochlor ESA, Acetochlor OA) from the tested water to levels below sanitary limits. As confirmed during long-term tests, the obtained activated carbon gradually loses its ability to remove the adsorbates of our interest over time. According to our findings, confirmed by pore size distribution analyses, the observed reduction in the pesticide removal efficiency is due in particular to the reduction of crucial for adsorption available mesopore volume. On this basis, we concluded that in the further steps of the process of developing the activated carbon, we must put much effort to obtain activated carbon showing features expected by customers, but even richer in mesopores. Moreover, assuming that the properties of the obtained activated carbon are satisfactory, but the presence of other substances, such as humic acids, competes with pesticides for adsorption sites, we plan to investigate the effect of the adsorbent during the long-term removal of pesticides from the coagulated and filtration water before it is directed to filtration on the bed activated carbon. We believe that the planned activities will result in obtaining both the procedure of industrial production of activated carbon intended for the removal of pesticides from the water, and recommendations for the customer on how to use the absorbent to obtain a good effect for a long time.

## Data Availability

The datasets used and/or analysed during the current study available from the corresponding author on reasonable request.
